# A Novel, Variance Component-Based Method for Detecting Brain-Behavior Associations in Neuroimaging Data

**DOI:** 10.1080/29979676.2025.2579919

**Published:** 2025-12-17

**Authors:** Christina E. Chen, Jeremy Rubin, Lior Rennert, Mackenzie Edmondson, Simon N. Vandekar, Russell T. Shinohara, for the Alzheimer’s Disease Neuroimaging Initiative

**Affiliations:** a Department of Biostatistics, Epidemiology, and Informatics, University of Pennsylvania, Philadelphia, PA; b Department of Public Health Sciences, Clemson University, Clemson, SC; c Merck & Co., Inc., Rahway, NJ; d Department of Biostatistics, Vanderbilt University, Nashville, TN

**Keywords:** Global and local inference, High-dimensional data, Variance component testing

## Abstract

The sequence kernel association test (SKAT) is a widely used and computationally efficient method in high-dimensional studies that tests for the joint effect of multiple predictors while accommodating covariates. However, the omnibus nature of the test hinders interpretation. We develop a new method called LaxKAT (linear maximum kernel association test) that can identify both global and local signal in high-dimensional data. The LaxKAT statistic maximizes the SKAT statistic over a pre-specified subspace of linear kernels. We demonstrate via simulations that it exhibits improved global and local power compared to previous methods while controlling the family-wise error rate (FWER). We also apply LaxKAT to neuroimaging data from cognitively normal controls in the Alzheimer’s Disease Neuroimaging Initiative (ADNI) to identify brain regions exhibiting sex-specific differences in cortical thickness patterns.

## 1. Introduction

Testing for global associations in high-dimensional data probes the overall relationship between a response and multiple predictors. For example, in genomics, a common objective involves testing for the association between a single phenotype and the allelic counts of different variants across the genome. In neuroimaging, considerable interest prevails in testing for the association between a single outcome and an imaging feature at different locations across the brain. Intuitively, detecting the cumulative influence of multiple predictors is easier than detecting the influence of a single predictor. However, in practice, determining the best way to aggregate information across predictors is challenging.

Moreover, localizing global signal is important for understanding biological mechanisms and selecting predictors to target for followup studies. Atlases that parcellate the cerebral cortex into multiple regions afford a flexible and standardized tool for tracking signal within morphological or functionally comparable locations. For example, cortical thinning occurs during both normal, aging-related and pathological processes such as Alzheimer’s disease (AD), but the trajectories and distributions of these changes differ (McDonald, McEvoy, and Gharapetian 2009). Thus, identifying the regions of cortical thinning most associated with mild cognitive disorder (MCI), a precursor to AD, can facilitate establishing biomarkers for detecting patients with increased risk of developing AD who would benefit from preventive treatments. This is particularly important because magnetic resonance imaging (MRI) is less invasive than acquiring CSF or PET biomarkers. Moreover, structural MRI measures different underlying processes compared to these methods (non-specific neurodegeneration vs. abnormal protein deposition) and thus provides complementary diagnostic information (Jack Jr. et al. 2016).

There are many methods that test for overall association, including the sum (of scores) test (Pan 2009), the sum of squared scores test (SSU, Pan 2009), the sum of powered scores test (SPU, Pan et al. 2014), the adaptive sum of powered scores test (aSPU, Pan and Shen 2011), the sequence kernel association test (SKAT, Wu et al. 2011), and optimal SKAT (SKAT-O, Lee et al. 2012), which were all developed originally for genomics data. The sum test and its variants, including the weighted scores test (WST, Wang and Elston 2007), are burden tests that perform well when most of the predictors are associated with the response and exert unidirectional effects. In contrast, SSU, SKAT, and their variants, including SPU, aSPU, and SKAT-O, are variance component tests and confer increased power when the predictors exert bidirectional effects. See Lee et al. (2014) for a comprehensive comparison of burden tests, variance component tests, their underlying models, and relative advantages. Recently, Vandekar, Reiss, and Shinohara (2019) proposed the projected scores test (PST), which maximizes Rao’s sum statistic (Rao 1948) over a pre-specified subspace of linear kernels. This maximization is equivalent to projecting the scores of the marginal tests onto a lower-dimensional subspace. Performing subsequent global and local inference on this subspace increases power by decreasing the number of degrees of freedom.

We adapt this idea by proposing LaxKAT (linear maximum kernel association test), which maximizes the SKAT statistic over a pre-specified subspace of linear kernels. Since variance component tests (such as SKAT) are known to be more powerful than burden tests (such as the sum test) in the setting of bidirectional signal, we hypothesize that LaxKAT will be more powerful than PST in a variety of settings.

The choice of subspace enables leveraging previous knowledge about the signal or testing hypotheses involving specific combinations of predictors. The latter application arises naturally in neuroimaging in the context of region-specific inference. Different atlases partition the brain into regions of interest (ROIs) according to anatomical or functional properties, and identifying the ROIs associated with a response can illuminate connections between biology and clinical outcomes. LaxKAT’s model accommodates these questions because an atlas is a subspace spanned by ROI masks.

In fact, the LaxKAT statistic simplifies in this setting to a maximum of scaled, ROI-specific SKAT statistics. In genomics, applying SKAT to candidate genes and then correcting for multiple comparisons enables refining the locations of causal variants (Wu et al. 2011). LaxKAT’s mathematical properties motivate adopting this idea to test for signal concentrated in ROIs via post hoc local inference. Thus, LaxKAT advances two central goals in analyzing high-dimensional neuroimaging data: it provides a more powerful way of conducting global inference and endorses a natural way of conducting local inference.

In Section 2, we derive the LaxKAT statistic. In Section 3, we conduct simulations to demonstrate that LaxKAT exhibits well-controlled family-wise error rate (FWER) as well as increased global and local power compared to currently available methods. We also apply LaxKAT to the Alzheimer’s Disease Neuroimaging Initiative (ADNI) dataset to identify regions where the cortical thickness patterns differ between male and female controls. In Section 4, we conclude by discussing LaxKAT’s applications and possible extensions.

## 2. Methods

Notation-wise, we will use bold, uppercase letters to denote matrices, bold, lowercase letters to denote vectors, and non-bold letters to denote scalars. Consider the generalized linear mixed model (GLMM)

(1) $$g(E[y_{i}|\beta ])=x_{i}^{⊤}c+z_{i}^{⊤}W\beta ,$$

where *g* denotes a link function, $y_{i}\in R$ denotes the response (ex. sex) of the *i*th subject, $x_{i}\in R^{m}$ denotes the nuisance covariates (ex. age) of the *i*th subject, $z_{i}\in R^{p}$ denotes the predictors (ex. cortical thickness across vertices) of the *i*th subject, $W=\text{diag}(w)\in R^{p\times p}$ denotes a *diagonal* weight matrix encoding the importance of each predictor, $\alpha \in R^{m}$ denotes the covariate effects, and $\beta \in R^{p}$ denotes the predictor effects.

Assume that α is fixed and β follows some distribution with first two moments E[β]=0 and $\text{var}[\beta ]=\tau^{2}\;I_{p}$, where $I_{p}$ denotes the p×p identity matrix. Note that each subject shares the same β, so this term does not require an index. Unlike the typical GLMM setup, our model specifies variability across predictors, not subjects. As in Wu et al. (2011), we have assumed for simplicity independent random effects, although more complex models allowing covariance between them are also possible.

We emphasize that the weight matrix W is pre-specified. It appears in the SKAT statistic (see Section 2.1) by conferring a weight to each predictor in the score calculation. Moreover, each term $w_{j}\beta_{j}$ in Wβ has variance $w_{j}^{2}\tau^{2}$, so introducing a weight matrix also allows flexibility around the assumption that all random effects share the same variance.

### 2.1. Review of SKAT

Wu et al. (2011) motivates SKAT by observing that testing the null hypothesis $H_{0}:\tau^{2}=0$ in (1) is equivalent to testing $H_{0}:\beta =0$. However, the former is preferable to testing $H_{0}:\beta =0$ in a fixed effects setting because it can be recast as a variance component score test, which is a locally most powerful test (Lin 1997).

Let $\hat{\alpha }$ denote the maximum likelihood estimate (MLE) of α under the covariate-only model. When *g* is a canonical link function, the estimated quasilikelihood score (Lin 1997) of $H_{0}:\tau^{2}=0$ equals

(2) $$\frac{1}{2}\{{\left(y-\hat{y}\right)}^{⊤}ZW^{2}Z^{⊤}(y-\hat{y})-\text{tr}[\Delta ZW^{2}Z^{⊤}]\},$$

where $y\equiv (y_{1},\ldots ,y_{n})\in R^{n}$ denotes the vector of responses, $Z\in R^{n\times p}$, the row-wise concatenation of $z_{1},\ldots ,z_{n}$, denotes the matrix of predictors, ${\hat{y}}_{i}\equiv g^{-1}(x_{i}^{⊤}\hat{\alpha })$ denotes the predicted response at $\alpha =\hat{\alpha }$ and β=0, and $\Delta =\text{diag}(\delta )\in R^{n\times n}$ denotes the diagonal matrix defined by $\delta_{i}\equiv {\left[g^{\prime }({\hat{y}}_{i})\right]}^{-1}$. We will focus on the normal GLMM for continuous responses and the Bernoulli GLMM for binary responses. In the former case, Δ simplifies to $I_{n}$, and in the latter case, $\delta_{i}={\hat{y}}_{i}(1-{\hat{y}}_{i})$.

SKAT omits the second term in (2), which is nonrandom for the normal GLMM and asymptotically negligible otherwise (Breslow and Clayton 1993; Zhang and Lin 2003), and defines the statistic

$$Q_{\text{SKAT}}\equiv {\left(y-\hat{y}\right)}^{⊤}ZW^{2}Z^{⊤}(y-\hat{y}).$$

It is straightforward to derive that the null distribution of $Q_{\text{SKAT}}$ is a linear combination of independent Chi-square distributions (Lin 1997; Wu et al. 2011). The SKAT software uses the Davies method (Davies 1990) to approximate its distribution function.

### 2.2. LaxKAT Statistic

A common choice for W in SKAT is a diagonal matrix with the *j*th diagonal entry determined by the estimated minor allele frequency (MAF) of the *j*th variant, reflecting the reasoning that rarer variants exert larger effects. No obvious parallel exists in neuroimaging because for many phenotypes, current knowledge does not justify any particular encoding that presupposes specific effect sizes across different brain locations. However, we can adapt the weighting framework to leverage prior knowledge about brain structure and function.

Specifically, we recognize that spatial organization within the brain predisposes to particular patterns of signal distribution. For example, we might hypothesize that our signal spans a subset of vertices delimited by ROI boundaries, that is, the optimal weighting matrix W equals a linear combination of ROI masks determined by a cortical atlas. This reasoning motivates maximizing the SKAT statistic over a pre-specified subspace of linear kernels to derive a new statistic, which we call the LaxKAT statistic.

More concretely, suppose that our atlas divides the cortex into *q* ROIs. Construct the subspace L of linear kernels generated by $\left\{B_{1},\ldots ,B_{q}\right\}$, where the diagonal of $B_{k}\in R^{p\times p}$ contains 1s in the coordinates indexing the vertices in the *k*th ROI and 0s elsewhere. Consider the statistic

(3) $$Q_{\text{LaxKAT}}\equiv \underset{W\in L}{\text{max}}\frac{{\left(y-\hat{y}\right)}^{⊤}ZW^{2}Z^{⊤}(y-\hat{y})}{\text{tr}[\Delta ZW^{2}Z^{⊤}]},$$

where the normalization follows naturally from the requirement that the statistic remains invariant to multiplying W by a scalar. Thus, the LaxKAT framework mitigates potential concerns about selecting one specific weight matrix, as SKAT requires, while still allowing additional insights about the structure of the optimal weights, by summarizing the information provided by weight matrices belonging to a subspace encoding this structure. Note that the LaxKAT statistic is related to the SKAT statistic in the same way that the PST statistic is related to the sum statistic in that the former equals the maximization of the latter over some subspace of linear kernels, up to normalization.

Even though (3) defines $Q_{\text{LaxKAT}}$ as a maximization problem, Theorem 2.1 shows that deriving this maximum does not require testing the infinitely many elements in L.

*Theorem 2.1*. If a *q*-dimensional subspace L of diagonal matrices in $R^{p\times p}$ admits a basis $\left\{B_{1},\ldots ,B_{q}\right\}$ of diagonal matrices such that $B_{k}B_{l}=0^{p\times p}$ for all k≠l (i.e., such that they contain mutually exclusive nonzero coordinates), then

$$Q_{\text{LaxKAT}}=\underset{1\leq k\leq q}{\text{max}}\frac{{\left(y-\hat{y}\right)}^{⊤}ZB_{k}^{2}Z^{⊤}(y-\hat{y})}{\text{tr}[\Delta ZB_{k}^{2}Z^{⊤}]}.$$

Proof. Note that any diagonal matrix W∈L admits a unique representation as a linear combination $\sum_{k=1}^{q}\phi_{k}B_{k}$ of the basis elements. Therefore, we obtain

$$\begin{array}{cc} Q_{\text{LaxKAT}} & =\underset{\phi \in R^{q}}{\text{max}}\frac{{\left(y-\hat{y}\right)}^{⊤}Z{\left(\sum_{k=1}^{q}\phi_{k}B_{k}\right)}^{2}Z^{⊤}(y-\hat{y})}{\text{tr}[\Delta Z{\left(\sum_{k=1}^{q}\phi_{k}B_{k}\right)}^{2}Z^{⊤}]}\\ & =\underset{\phi \in R^{q}}{\text{max}}\frac{\sum_{k=1}^{q}\phi_{k}^{2}{\left(y-\hat{y}\right)}^{⊤}ZB_{k}^{2}Z^{⊤}(y-\hat{y})}{\sum_{k=1}^{q}\phi_{k}^{2}\;\text{tr}[\Delta ZB_{k}^{2}Z^{⊤}]}\\ & =\underset{\phi \in R^{q}}{\text{max}}\sum_{k=1}^{q}\frac{\phi_{k}^{2}}{\sum_{k=1}^{q}\phi_{k}^{2}}\;\frac{{\left(y-\hat{y}\right)}^{⊤}ZB_{k}^{2}Z^{⊤}(y-\hat{y})}{\text{tr}[\Delta ZB_{k}^{2}Z^{⊤}]}\\ & =\underset{1\leq k\leq q}{\text{max}}\frac{{\left(y-\hat{y}\right)}^{⊤}ZB_{k}^{2}Z^{⊤}(y-\hat{y})}{\text{tr}[\Delta ZB_{k}^{2}Z^{⊤}]}, \end{array}$$

where the last step follows from the observation that the maximum of a weighted sum of positive terms equals the maximum of the individual terms. ▪

In this article, we focus on brain atlases and consider only subspaces generated by ROI-specific masks. This restriction implies probing the space of weight matrices that weight all vertices within an ROI equally, although their effects may vary due to the random effects model. This is a reasonable working assumption because vertices occupying the same anatomical region often share functional properties and thus affect responses similarly.

However, LaxKAT’s central insight transfers to more complex signal spatial distributions. Other examples of subspaces satisfying the condition in Theorem 2.1 include the subspace generated by the columns of the components matrix produced by orthogonal nonnegative matrix factorization (ONNMF), a variation of nonnegative matrix factorization (NNMF) that enforces orthogonal components to encourage clustering (Ding et al. 2006). Sotiras, Resnick, and Davatzikos (2015) showed that applying NNMF to neuroimaging data extracts sparse and contiguous components that recover interpretable structural and functional units. These units, which denote portions of the brain that co-vary across individuals, potentially share underlying biological processes, and probing their associations with disease phenotypes can reveal additional information about disease pathophysiology.

In fact, it is straightforward to compute the LaxKAT statistic even for subspaces that do not admit a basis of the form described in Theorem 2.1. For example, Vandekar, Reiss, and Shinohara (2019) proposes that the signal might concentrate in a subspace generated by the first few principal components (i.e., axes of maximal variation) across brain vertices. Theorem 8.1 shows that computing $Q_{\text{LaxKAT}}$ in the general case entails solving a generalized eigenvalue problem, for which efficient algorithms exist (Moler and Stewart 1973).

### 2.3. LaxKAT for Global and Local Inference

No closed-form formula exists for the cumulative density function of the maximum of correlated SKAT statistics. Nevertheless, we may obtain, via permutations, the null distributions of both $Q_{\text{LaxKAT}}$ and the scaled, ROI-specific SKAT statistics

(4) $$Q_{k}\equiv \frac{{\left(y-\hat{y}\right)}^{⊤}ZB_{k}^{2}Z^{⊤}(y-\hat{y})}{\text{tr}[\Delta ZB_{k}^{2}Z^{⊤}]},$$

where each $B_{k}$ denotes an ROI mask. For a continuous response, this involves permuting the residuals $y_{i}-{\hat{y}}_{i}$ and recomputing the statistics for each permutation. For a binary response, this involves permuting both the residuals and the predicted responses for each permutation. In either case, the procedure tests the desired null hypothesis because the residuals and predicted values absorb the correlation between the response and nuisance covariates (Winkler et al. 2014).

To test for local association, we obtain a *p*-value for each ROI by comparing $Q_{k}$ to its null distribution and correct for multiple comparisons via the Holm method (Holm 1979). In the case of a normal response, $Q_{k}$ equals a SKAT statistic divided by a constant, so it is straightforward to compute its *p*-value according to the method in Wu et al. (2011). In the case of a binary (or arbitrarily distributed) response, when the denominator in (4) becomes random, it is straightforward to derive a *p*-value for the *k*th ROI according to the permutation distribution of $Q_{k}$ recoverable from the permutations generated during the computation of $Q_{\text{LaxKAT}}$’s *p*-value.

Although the decoupling of the LaxKAT statistic into ROI-specific (scaled) SKAT statistics and the consequent correspondence between the global and post hoc local tests follow naturally from the mathematical properties of atlases, we caution against one potential misinterpretation. A significant global test rejects the null hypothesis, as specified in Section 2.1, that none of the vertices are associated with the response. Importantly, it does not localize the signal to only the ROI producing the maximum. Thus, local tests succeeding a significant global test can detect none, one, or multiple significant ROIs.

### 2.4. Simulations

To assess the performance of our proposed test under realistic neuroimaging conditions, we used data from the Alzheimer’s Disease Neuroimaging Initiative (ADNI), described in Jack Jr. et al. (2008) and Petersen et al. (2010). The ADNI is a longitudinal study enrolling both healthy controls and patients with mild cognitive impairment (MCI) and Alzheimer’s disease (AD). We followed the exclusion criteria in Vandekar, Reiss, and Shinohara (2019) and used three-dimensional T1-weighted structural MRIs from n=399 patients who have been diagnosed with MCI and with composite memory scores available. We used the Desikan-Killiany (DK) atlas (Desikan et al. 2006), a cortical parcellation comprising 34 ROIs in the right hemisphere. For our predictors, we considered the cortical thickness at p=9212 vertices on the right hemisphere, estimated using FreeSurfer (Dale, Fischl, and Sereno 1999; Fischl and Dale 2000). We obtained this set of vertices by excluding from the original 10,242 right hemisphere vertices in fsaverage5 space (Fischl et al. 1999) the unassigned vertices, the vertices in the corpus callosum, and those vertices containing 0s or NAs among our n=399 subjects. Thus, our original image data is a matrix $Z_{0}\in R^{n\times p}$ of cortical thickness values. Note that the vertices that remain unassigned post parcellation constitute only a small proportion (8.1%) of the cortical surface. For our covariates, we included age and sex (as well as an intercept term).

In our simulations, we assessed LaxKAT’s performance under four different scenarios. For our first experiment, we concentrated our signal on two ROIs: the banks of the superior temporal sulcus and the caudal anterior cingulate cortex on the right hemisphere. (For simplicity, we chose the two ROIs in the atlas with names appearing first in alphabetical order.) For each level of signal strength $\tau^{2}$ (varying from 0 to 0.01), we generated 1000 outcomes vectors according to $y∼N(0,I_{n})$. For each y, we generated effect sizes $\gamma ∼N(0,\tau^{2}\;I_{p})$ and perturbed our data matrix according to

(5) $$Z=Z_{0}+y⊗(V\gamma ),$$

where V denotes the diagonal matrix containing 1s in the coordinates indexing vertices in the signal ROIs and 0s elsewhere and ⊗ denotes the outer product. Thus, the *j*th column of Z equals the *j*th column of $Z_{0}$ plus $\gamma_{j}y$, where the latter term equals 0 unless the *j*th vertex belongs to a signal ROI.

For our second experiment, we concentrated our signal on five ROIs (again, according to alphabetical order): the banks of the superior temporal sulcus, the caudal anterior cingulate cortex, the caudal division of the middle frontal gyrus, the cuneus cortex, and the entorhinal cortex. For each level of signal strength $\tau^{2}$, we generated 1000 iterations of (y,β,Z) as in the first experiment.

For our third experiment, we concentrated our signal on the same five ROIs as in the second experiment. For each level of signal strength $\tau^{2}$ (varying from 0 to 0.01), we generated 1000 outcomes vectors as in the first experiment. For each outcomes vector, we generated a perturbed data matrix according to (5), where $\gamma =\tau^{2}\;1$ (a constant vector of $\tau^{2}$ s).

For our fourth experiment, we concentrated our signal on the same five ROIs as in the second experiment. For each level of signal strength $\tau^{2}$ (varying from 0 to 0.1), we generated 1000 continuous outcomes vectors according to $y_{0}=(y_{0,\;1},\ldots ,y_{0,\;n})∼N(0,I_{n})$. For each $y_{0}$, we generated effect sizes $\gamma ∼N(0,\tau^{2}\;I_{p})$ and perturbed our data matrix according to

(6) $$Z=Z_{0}+y_{0}⊗(V\gamma ).$$

We then transformed each $y_{0}$ via

$$y_{i}\equiv I\left\{\frac{\;\text{exp}\;(y_{0,\;i})}{1+\;\text{exp}\;(y_{0,\;i})}>0.5\right\},$$

where I{·} denotes the indicator function, to obtain a binary outcomes vector $y\equiv (y_{1},\ldots ,y_{n})$.

For each simulated outcomes vector and perturbed data matrix (1000 pairs for each of the four experiments), we tested for global and ROI-wise association between y and Z using LaxKAT. For the former, we compared LaxKAT to SKAT and PST. For the latter, we compared LaxKAT to a more classical, marginal (mass-univariate) analysis approach, in which we regressed, for each ROI, the response onto age, sex, and the ROI-specific average cortical thickness and tested for a nonzero coefficient for average thickness using a Wald test.

Note that in all of our experiments, the steps in (5) and (6) ensure that our response is associated with only the vertices in the signal ROIs, so that we may evaluate the local power and false discovery rate (FDR) of our methods in a setting without spurious associations induced by the correlation structure among vertices. This contrasts with the typical setup of simulating a random effects vector and then generating the outcomes vector from its interaction with the fixed image matrix. Thus, we have used γ in our description instead of β to emphasize that the relationship here between our response and predictors deviates from the relationship between y and Z in (1). However, this slight discrepancy actually encourages stronger conclusions because our experiments evaluate LaxKAT’s ability to detect local and global signal even when the simulation model does not coincide with its underlying statistical model.

## 3. Results

### 3.1. Simulation Results

**Figure 1:**
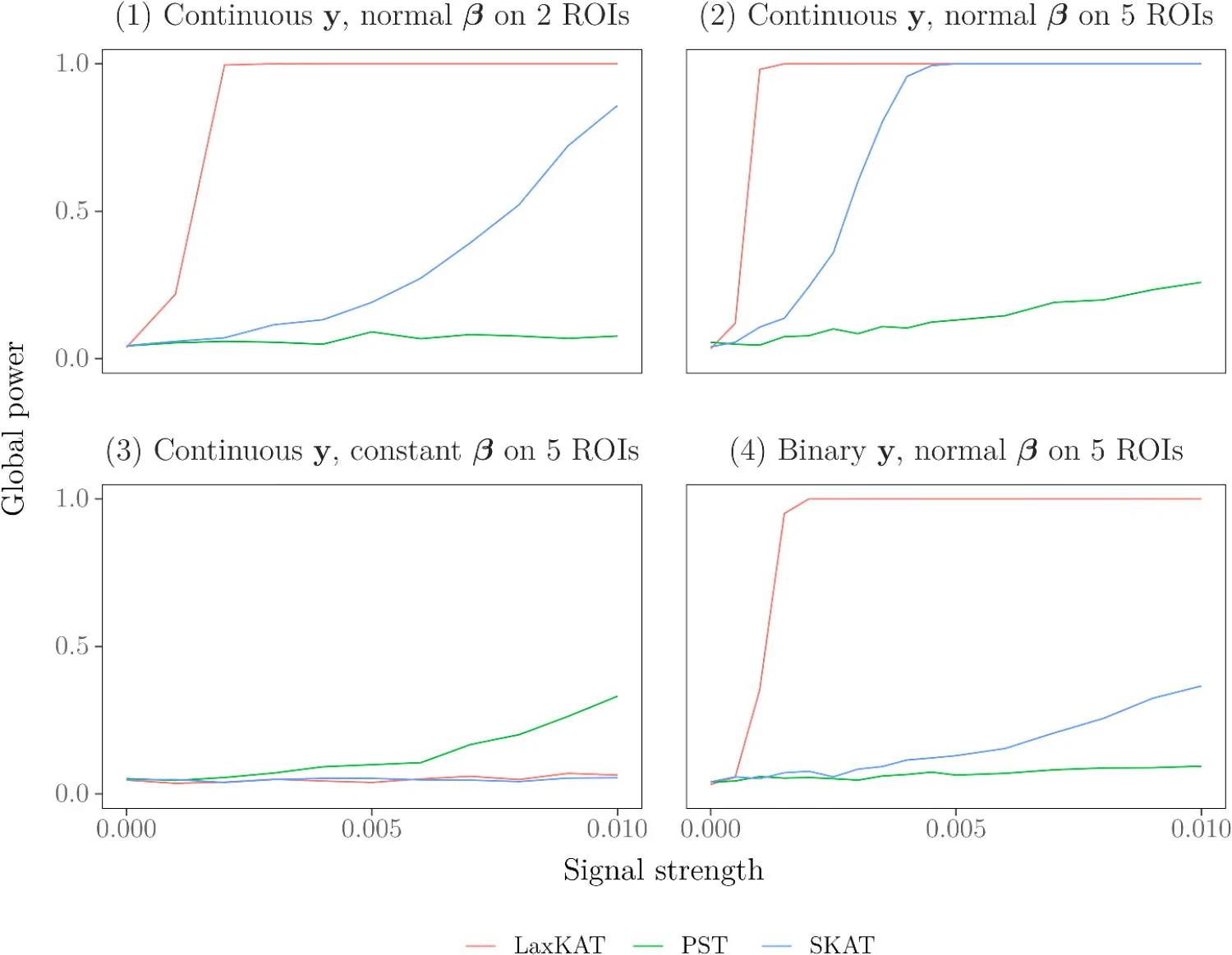
Global power comparison for LaxKAT, SKAT, and PST across a range of simulation settings. Top left: continuous response and normally distributed signal on two ROIs. Top right: continuous response and normally distributed signal on five ROIs. Bottom left: continuous response and constant signal on five ROIs. Bottom right: binary response and normally distributed signal on five ROIs.

To assess the global power of LaxKAT, we counted for each value of $\tau^{2}$ the proportion of simulations (out of 1000) producing a global *p*-value less than 0.05. See Figure 1 for plots of LaxKAT’s global power compared to SKAT and PST in our four simulation scenarios. Note that in all four plots, the curve corresponding to LaxKAT intersects the *y*-axis ($\tau^{2}=0$) at approximately 0.05, which indicates well-controlled Type I error rate. In the first, second, and fourth experiments (top left and right and bottom right panels in Figure 1), LaxKAT significantly outperforms the other methods across all signal levels. In the third experiment (bottom left panel in Figure 1), PST slightly outperforms both LaxKAT and SKAT, which is expected because the constant signal matches the model assumptions of PST. Nevertheless, the comparable performances of LaxKAT and SKAT in this extreme setting, together with the observation that the atlas subspace surveyed by LaxKAT includes the identity SKAT kernel, suggest that even under model misspecification, LaxKAT affords an attractive alternative to SKAT in detecting deviations from the null.

In reality, predicting LaxKAT’s performance relative to other methods depends on multiple factors, including the distribution of the signal and the relationship between the signal and the covariance structure among the predictors themselves, which can be probed via exploratory analyses such as vertex-wise association tests. Moreover, the scaling term in the definition of the LaxKAT statistic precludes any straightforward comparison with SKAT. We refer readers to Vandekar, Reiss, and Shinohara (2019) for simulation results comparing PST to popular SKAT variants (aSPU, etc.) to further contextualize LaxKAT within the diverse landscape of global signal detection methods for high-dimensional data. Despite these challenges, our results demonstrate that LaxKAT can match or exceed currently available methods in experiments simulating a range of signal shape, strength, and sparsity.

**Figure 2:**
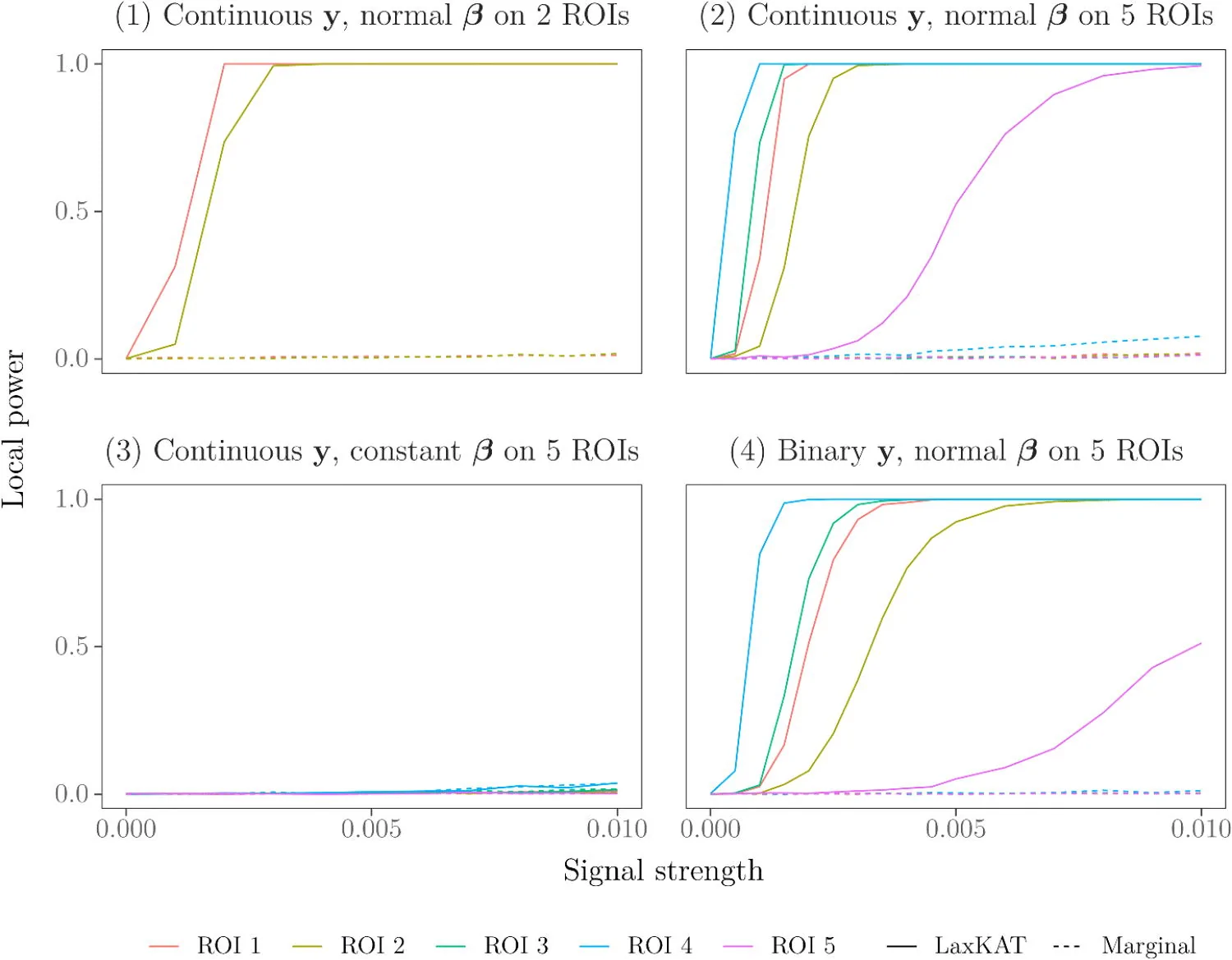
Local power comparison for LaxKAT and marginal association tests at different signal levels. The dotted lines correspond to the baseline method. For conciseness, we have labeled the ROIs with numbers 1–5, corresponding to the order in which we listed them in Section 2.4.

To assess the local power of LaxKAT, we counted for each (out of two or five, depending on the experiment) signal ROI the proportion of simulations (out of 1000) producing a multiplicity-corrected, ROI-specific *p*-value not exceeding 0.05. See Figure 2 for plots of LaxKAT’s local power compared to the baseline method described in Section 2.4. Note that in all four plots, the curve corresponding to LaxKAT intersects the *y*-axis ($\tau^{2}=0$) below 0.05. This indicates well-controlled FWER, which is consistent with the definition of the LaxKAT statistic as the maximum of ROI-specific statistics. Moreover, LaxKAT performs significantly better than mass-univariate regression in the case of normally distributed signal (experiments 1, 2, 4) and similarly to mass-univariate regression in the case of constant signal (experiment 3). Again, LaxKAT’s lower power in the third experiment compared to the other experiments is expected because the constant signal simulation deviates from the model assumptions of LaxKAT and SKAT. However, the baseline method’s comparably low power under constant signal, even though this presents an ideal scenario for burden tests (that, e.g., collapse vertices within ROIs linearly by averaging their cortical thickness), suggests that even under model misspecification, mass-univariate regression does not outperform LaxKAT significantly across some range of signal strength.

**Figure 3:**
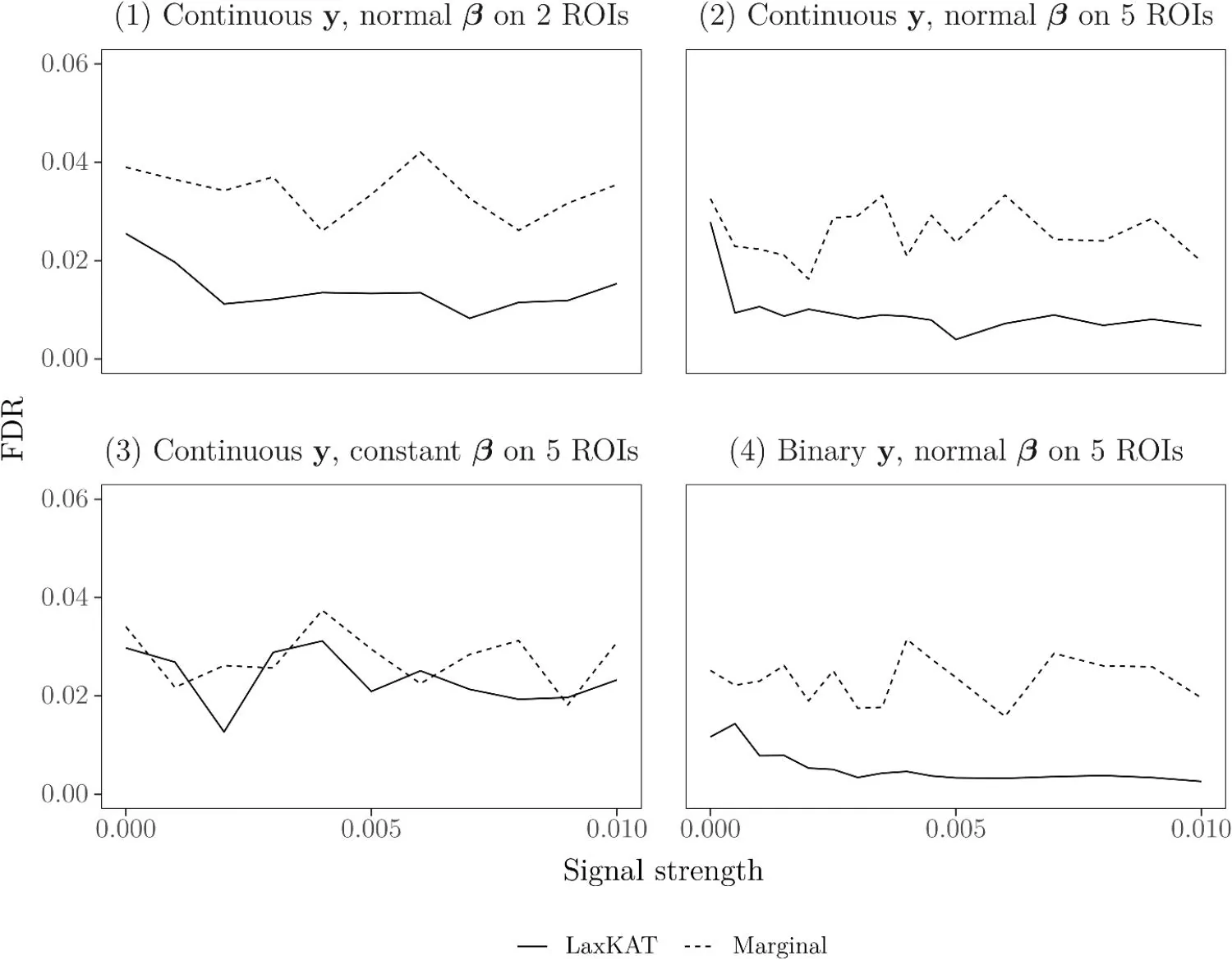
FDR comparison for LaxKAT and marginal association tests at different signal levels. The dotted lines correspond to the baseline method. Each point represents the average FDR over 1000 simulations.

We also assessed the FDR of LaxKAT and the baseline (mass-univariate regression) method by computing for each simulation the proportion of false discoveries among the ROIs where we rejected the null hypothesis. See Figure 3 for plots of LaxKAT’s FDR compared to mass-univariate regression, where each point represents the average FDR over 1000 simulations. Note that LaxKAT exhibits lower FDR compared to mass-univariate regression in the case of normally distributed signal and similar FDR in the case of constant signal. Taken together, the results in Figures 2 and 3 indicate that LaxKAT performs at least as well as mass-univariate regression both in identifying the correct signal ROIs and in rejecting the non-signal ROIs under different signal shapes.

### 3.2. Application to ADNI

We applied LaxKAT to the ADNI dataset (see description in Section 2.4) to understand sex-specific differences in cortical thickness among cognitively normal controls. Many previous studies have detected differences in overall and regional cortical thickness between men and women (Good et al. 2001; Verchinski et al. 2000; Luders et al. 2004; Sowell et al. 2006). Identifying the brain regions most strongly associated with this difference can link biological and clinical sex differences.

**Figure 4:**
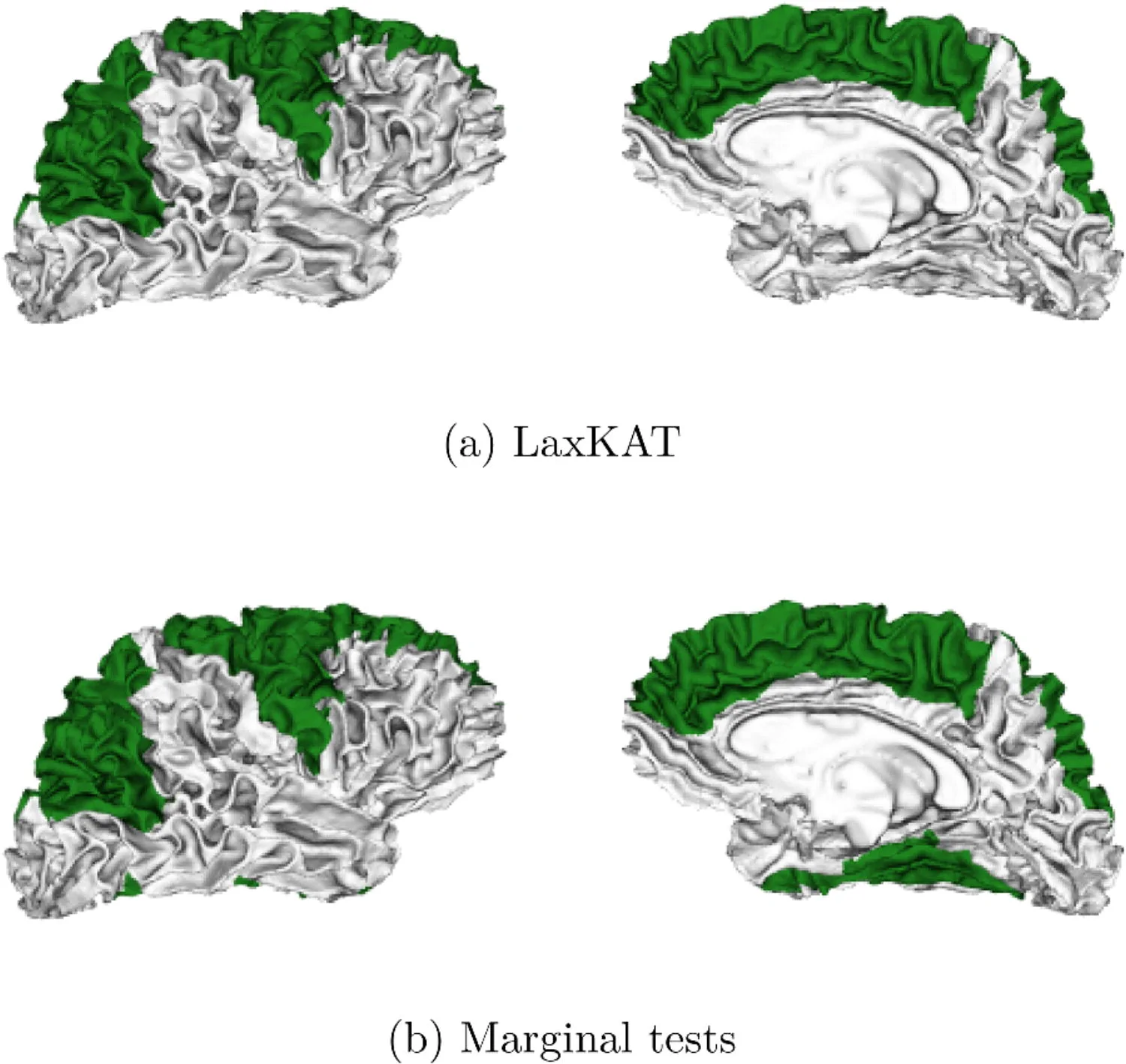
Right hemisphere ROIs from the DK atlas identified by LaxKAT versus marginal tests to exhibit sex-specific differences in cortical thickness among ADNI controls.

In this experiment, we performed LaxKAT using a logistic model and the DK atlas, regressing sex against age, memory scores, and cortical thickness measurements on the 229 controls from Vandekar, Reiss, and Shinohara (2019) (and the same p=9,212 vertices in the right hemisphere). For global inference, both LaxKAT and SKAT detected significant global associations. For local inference, marginal tests on average regional cortical thickness detected six significant ROIs in the right hemisphere, while LaxKAT detected two additional ROIs, the parahippocampal gyrus and the fusiform gyrus. Table 1 lists the eight ROIs identified by LaxKAT, ordered by their *p*-values post multiplicity correction. Figure 4 provides a visualization of the significant regions identified by the two methods.

**Table 1:** Right hemisphere ROIs from the DK atlas identified by LaxKAT to exhibit sex-specific differences in cortical thickness among ADNI controls.

Region	Lobe	LaxKAT	Marginal
Caudal division of the middle frontal gyrus	Frontal	0.0034	0.0015
Superior frontal gyrus	Frontal	0.0034	0.0027
Superior parietal cortex	Parietal	0.0034	0.0032
Inferior parietal cortex	Parietal	0.0062	0.0118
Parahippocampal gyrus	Temporal	0.0090	0.1077
Precentral gyrus	Frontal	0.0174	0.0326
Fusiform gyrus	Temporal	0.0336	0.1250
Paracentral lobule	Frontal	0.0351	0.0495

The results indicate that regions exhibiting sex differences span the frontal, temporal, and parietal lobes. Notably, the regions yielding the lowest *p*-values are the caudal division of the middle frontal gyrus, superior frontal gyrus, and superior parietal cortex. These findings corroborate previous studies that have targeted similar questions with different methods (Im et al. 2006). Moreover, the concentration of these significant regions in the frontal, temporal, and parietal lobes align with previous results linking gray matter volume to behavioral and cognitive sex differences (Schlaepfer et al. 1995; Yurgelun-Todd, Killgore, and Young 2002). Finally, LaxKAT’s detection of the fusiform gyrus and parahippocampal cortex, both omitted by the marginal tests, additionally suggests sex differences in object perception and recognition or memory and demonstrates the potential power gains by leveraging regional spatial information.

## 4. Discussion

In this work, we have developed a new method that maximizes a scaled version of the SKAT statistic over a pre-specified subspace of linear kernels and enables testing for both global and ROI-specific associations in high-dimensional neuroimaging data. This method, which we refer to as LaxKAT, controls the FWER while also improving power over currently available methods in a variety of realistic neuroimaging settings.

Moreover, LaxKAT is computationally efficient. Computing the LaxKAT statistic itself is fast because it reduces to deriving a maximum or solving a generalized eigenvalue problem (for which LAPACK-based functions exist in R), and computing its *p*-value is fast because our implementation parallelizes permutations. For example, applying LaxKAT to each simulated dataset (n=399 subjects, p=9,212 vertices) in our experiments requires less than 2 min on 100 CPU cores.

This article emphasizes the utility of applying LaxKAT to subspaces generated by atlases to probe region-specific questions, but the theory underlying the global LaxKAT statistic pertains to any subspace (see Theorem 8.1). This subspace, which we specify a priori, determines LaxKAT’s results and their interpretation. Therefore, implementing LaxKAT effectively predicates on selecting carefully a subspace that either incorporates previous knowledge about the signal or matches our specific questions.

In terms of limitation, our proposed method of post hoc local inference can fail to identify any signal region despite the detection of global association. Moreover, for atlases, the decoupling of the LaxKAT statistic into ROI-specific terms motivates ROI-wise hypothesis testing (i.e., hypothesis testing on the basis elements of the subspace). However, for a general subspace such as the subspace generated by the top several principal component (PC) loadings, conducting vertex-wise testing becomes more natural, although this would require a different approach. In the maximization problem defining the PST statistic, the maximum is related to the projection of the vector of marginal scores onto the pre-specified subspace (Vandekar, Reiss, and Shinohara 2019). The LaxKAT statistic does not admit such a geometric interpretation, but we may borrow this idea by viewing the linear kernel generating the LaxKAT statistic as a change-of-basis matrix and conducting post hoc inference on a transformed set of scores. However, this generalization will entail more extensive theoretical and empirical exploration.

Even in the case of atlases, LaxKAT can identify omnibus, but not local, effects within ROIs. A multi-level extension of LaxKAT could potentially address this deficiency. For example, for higher-resolution atlases such as the Schaefer parcellations (Schaefer et al. 2018), whose ROIs cluster into functional networks, we suggest a two-step LaxKAT test that first localizes significant networks and then localizes sub-regions within specific networks. This approach might also befit scenarios where more than one candidate subspace appears reasonable but testing their direct sum is unappealing.

Finally, we note that variance component tests and their corresponding variance component score statistics have already been derived for random effects models more general than the one we assumed in Section 2. Specifying a more complex covariance matrix for the vector of random effects can accommodate, for example, longitudinal and clustered data (Lin 1997). This connection between variance component tests and LaxKAT also motivates studying variance component testing in models that are not GLMMs, such as the Cox proportional hazards model, to enable identifying brain regions associated with disease progression. Thus, we envision exploiting this idea of maximizing a score statistic over a subspace to develop LaxKAT into a flexible method that can detect global and local signal in the brain in a way that incorporates not only spatial structure within the brain but also group structure within samples.

## Data Availability

Data used in preparation of this article were obtained from the Alzheimer’s Disease Neuroimaging Initiative (ADNI) database (http://adni.loni.usc.edu).

## Software

Code for LaxKAT is available at https://github.com/christinachen94/LaxKAT.

## Appendix

*Theorem 8.1*. Let $\left\{B_{1},\ldots ,B_{q}\right\}$ denote a basis for a *q*-dimensional subspace L of q×q diagonal matrices. Retain the setup of (1). Then $Q_{\text{LaxKAT}}$ as defined in (3) equals the largest eigenvalue solving the generalized eigenvalue problem Nϕ=λDϕ, with $N,D\in R^{q\times q}$ defined by

$$N_{kl}={\left(y-\hat{y}\right)}^{⊤}ZB_{k}B_{l}Z^{⊤}(y-\hat{y})$$

and

$$D_{kl}=\text{tr}[\Delta ZB_{k}B_{l}Z^{⊤}].$$

Proof. For any W∈L, write $W=\sum_{k=1}^{q}\phi_{k}B_{k}$. Then

$$ZW^{2}Z^{⊤}=Z{\left(\sum_{k=1}^{q}\phi_{k}B_{k}\right)}^{2}Z^{⊤}=\sum_{k,\;l}\phi_{k}\phi_{l}ZB_{k}B_{l}Z^{⊤},$$

so

$$\begin{array}{cc} {\left(y-\hat{y}\right)}^{⊤}ZW^{2}Z^{⊤}(y-\hat{y}) & =\sum_{k,\;l}\phi_{k}\phi_{l}{\left(y-\hat{y}\right)}^{⊤}ZB_{k}B_{l}Z^{⊤}(y-\hat{y})\\ & =\phi^{⊤}N\phi \end{array}$$

and

$$\text{tr}[\Delta ZW^{2}Z^{⊤}]=\sum_{k,\;l}\phi_{k}\phi_{l}\;\text{tr}[\Delta ZB_{k}B_{l}Z^{⊤}]=\phi^{⊤}D\phi .$$

Therefore,

$$Q_{\text{LaxKAT}}=\underset{\phi \in R^{q}}{\text{max}}\frac{\phi^{⊤}N\phi }{\phi^{⊤}D\phi },$$

the maximum of a generalized Rayleigh quotient. It is a well-known result (Courant and Hilbert 1953) that the maximum value and the value of ϕ achieving this maximum equal the largest eigenvalue and its corresponding eigenvector solving the generalized eigenvalue problem Nϕ=λDϕ. Note that the maximum is invariant to the choice of basis for L because if $\left\{B_{1}^{\prime },\ldots ,B_{q}^{\prime }\right\}$ is another basis for L, with invertible change-of-basis matrix $M\in R^{q\times q}$, that is, such that

$$B_{k}^{\prime }=\sum_{l=1}^{q}m_{kl}B_{l}\;\forall 1\leq k\leq q,$$

then

$$\begin{array}{cc} N_{kl}^{\prime } & \equiv {\left(y-\hat{y}\right)}^{⊤}ZB_{k}^{\prime }B_{l}^{\prime }Z^{⊤}(y-\hat{y})\\ & ={\left(y-\hat{y}\right)}^{⊤}Z(\sum_{r=1}^{q}m_{\text{kr}}B_{r})(\sum_{r=1}^{q}m_{lr}B_{r})Z^{⊤}(y-\hat{y})\\ & =\sum_{r,\;s}m_{\text{kr}}m_{ls}{\left(y-\hat{y}\right)}^{⊤}ZB_{r}B_{s}Z^{⊤}(y-\hat{y})\\ & =\sum_{r,\;s}m_{\text{kr}}m_{ls}N_{\text{rs}}, \end{array}$$

so $N^{\prime }=M^{⊤}NM$ and likewise $D^{\prime }=M^{⊤}DM$. Thus, the generalized eigenvalue problems generated by the two bases share the same generalized eigenvalues. ▪
